# Barriers and facilitators to integrating care: experiences from the English Integrated Care Pilots

**DOI:** 10.5334/ijic.982

**Published:** 2012-07-24

**Authors:** Tom Ling, Laura Brereton, Annalijn Conklin, Jennifer Newbould, Martin Roland

**Affiliations:** RAND Europe, Westbrook Centre, Milton Road, Cambridge, CB4 1YG, UK and Currently Head of Impact, Innovation and Evidence, Save the Children, 1 St John's Lane, London, EC1M 4AR, UK; RAND Europe, Westbrook Centre, Milton Road, Cambridge, CB4 1YG, UK; RAND Europe, Westbrook Centre, Milton Road, Cambridge, CB4 1YG, UK; RAND Europe, Westbrook Centre, Milton Road, Cambridge, CB4 1YG, UK; Professor of Health Services Research, Cambridge Centre for Health Services Research, Institute of Public Health, University of Cambridge, Forvie Site, Robinson Way, Cambridge, CB2 0SR, UK

**Keywords:** integrated care, facilitators, barriers, England

## Abstract

**Background:** In 2008, the English Department of Health appointed 16 ‘Integrated Care Pilots’ which used a range of approaches to provide better integrated care. We report qualitative analyses from a three-year multi-method evaluation to identify barriers and facilitators to successful integration of care.

**Theory and methods:** Data were analysed from transcripts of 213 in-depth staff interviews, and from semi-structured questionnaires (the ‘Living Document’) completed by staff in pilot sites at six points over a two-year period. Emerging findings were therefore built from ‘bottom up’ and grounded in the data. However, we were then interested in how these findings compared and contrasted with more generic analyses. Therefore after our analyses were complete we then systematically compared and contrasted the findings with the analysis of barriers and facilitators to quality improvement identified in a systematic review by Kaplan et al. (2010) and the analysis of more micro-level shapers of behaviour found in Normalisation Process Theory (May et al. 2007). Neither of these approaches claims to be full blown theories but both claim to provide mid-range theoretical arguments which may be used to structure existing data and which can be undercut or reinforced by new data.

**Results and discussion:** Many barriers and facilitators to integrating care are those of any large-scale organisational change. These include issues relating to leadership, organisational culture, information technology, physician involvement, and availability of resources. However, activities which appear particularly important for delivering integrated care include personal relationships between leaders in different organisations, the scale of planned activities, governance and finance arrangements, support for staff in new roles, and organisational and staff stability. We illustrate our analyses with a ‘routemap’ which identifies questions that providers may wish to consider when planning interventions to improve the integration of care.

## Introduction

Healthcare has improved greatly over the past 20 years partly due to an increased focus on evidence-based medicine (EBM) and increased specialist input focused on single diseases. This can lead to fragmentation of patient care, especially for the growing number of people with multiple chronic conditions [1]. Fragmentation can occur both within and between care sectors, for example, through structural and financial barriers dividing providers at the interfaces of primary and secondary care, or between health and social care [2]. This fragmentation creates wider system-level inefficiencies, and a policy focus in many countries has changed in recent years to providing whole-patient care through more integrated approaches [3, 4]. It is suggested that better-integrated delivery will both improve the quality and reduce the cost of health care, and ultimately improve health outcomes [5].

In response to the perceived need to provide better integrated care, the Department of Health in England held a national competition in 2008 to appoint ‘Integrated Care Pilots’ [6, 7]. There was no blueprint for proposals: rather the government was seeking ‘bottom-up’ innovative approaches to providing better integrated care. Sixteen sites were appointed across the country which targeted a range of client groups, most commonly elderly people with multiple co-morbidities. Other sites focused on people with diabetes, cardiovascular disease and substance abuse. Interventions included variations of case management, multidisciplinary team working, and the development of new organisational structures to support integration. The focus of the sites reported in this paper is predominantly on closer integration between primary care, community care and social care, in contrast to integration between primary and secondary care, which has been the focus of integration in other countries [8]. The interventions introduced by the sixteen sites are described in more detail elsewhere [9]. The research reported here is part of the national evaluation of all sixteen Integrated Care Pilots [10].

Reviews of other integrated care initiatives show that their effectiveness, and the factors that facilitate or impede success, depend substantially on the context in which the intervention takes place [11]. Attempts to integrate care cannot therefore be seen separately from their clinical, geographic, financial and policy contexts. In this paper we review the barriers and facilitators to delivering integrated services as perceived by staff members involved with the English Integrated Care Pilots and relate these to two current theories of organisational change. In conclusion, we suggest a ‘routemap’ that summarises the issues which need to be considered by decision-makers in delivering better integrated care.

## Method

### Data collection

An overall account of the mixed methods evaluation of the Integrated Care Pilots has been published previously in this journal [12]. Key elements of the qualitative data collection and analysis are outlined here.

First, a common template (the ‘Living Document’) was developed for semi-structured data collection from all sixteen pilot sites and completed on six occasions at approximately six monthly intervals during the course of the project, including one just before and one after the end of the formal two-year pilot period. A lead staff member at each site, most often the project manager, collated and entered information from a wide range of staff involved in the pilot. The template was organised around a series of broad questions, which allowed staff to contribute views on what had facilitated or prevented progress to date.

Second, 213 semi-structured interviews were conducted with staff in a smaller number of pilots selected for in-depth case study analysis at the start (six sites) and one year into the pilot period (four sites). These interviews included both clinicians and managers of various levels of seniority, and focussed on staff experience of delivering care, the apparent impacts on patient care, interactions with other professional groups and organisations, and perceived implications for the wider care system. Each interview lasted approximately one hour and was audio-recorded, transcribed and coded using NVivo. In addition, data were included from a number of staff meetings, with field notes made by researchers who acted as non-participant observers.

### Analysis

Coding of interview data was carried out by AC, JN, LB and two other members of RAND Europe staff who first coded a sample of data, met to review the results and then recoded with a revised codebook. This process was then repeated to produce a codebook for final coding. Throughout the analysis, additional queries about coding were discussed by the group to resolve any uncertainties or differences in approach and additional codes added where agreed. Living Documents from all 16 sites were reviewed by the same researchers with a focus on the barriers and facilitators to pilot success as reported by staff. The themes we identified in the analysis of the interview and Living Document data were allowed to emerge from the data rather than being based on predetermined categories.

Due to the short time scale of the pilot programme, analysis of the data sources focussed on understanding staff opinions on what had helped or hindered them in establishing their pilot programme, as opposed to how barriers and facilitators changed during the course of the pilot period. Data synthesis built on a narrative approach [13] used thematic analysis for categorising data. In analysing findings from both the living documents and interviews, we categorised the data according to themes common to organisational change: namely, the nature of the intervention, professional factors, social factors, organisational factors and financial factors.

Based on our results, we then compared our findings to those presented by Kaplan et al. [14] in a comprehensive systematic review of barriers and facilitators to quality improvement activities; and also related our results to May et al.’s Normalisation Process Model [15, 16]. We aimed to understand the barriers and facilitators identified in terms of those likely to be particularly relevant to the development of integrated care and those likely to be generic to organisational change more broadly.

## Results

Barriers and facilitators were often two sides of the same coin (e.g., good management/poor management) and so the issues identified are presented here by theme rather than attempting to identify barriers and facilitators separately.

### Structure and characteristics of organisations and interventions

#### Size and complexity of the intervention

For most sites, the size and complexity of the intervention was important in determining how much progress was achieved during the pilot period. This picture was further complicated by lead organisations not always being in control of the full range of planned activity. How well the lead organisations supplemented direct control with indirect methods of producing change often shaped how the implementation proceeded.

Pilots whose interventions included multiple components (some had up to ten separate workstreams) reported greater challenges of managing change, and they were often greater than they had anticipated. In contrast, sites introducing simple, single-faceted interventions made more rapid progress. For example, a site running a highly focused falls prevention service described the benefit of having management concentrated around ‘a small central team’ with the ability and authority to come to quick decisions and drive the project forward. In contrast, where multiple interventions were attempted and many partners were involved, sites reported that the complexity of proposed work made it difficult to communicate the details of the intervention to all parties and identify the role of each participant group. These results do not imply that large-scale complex activities should be avoided, rather they suggest that substantial time and resources need be available to ensure that mutual understanding extends beyond the core project team and to ensure clear allocation of tasks. This was especially important where the aims were more ambitious, aiming to transform the way care was delivered.

Multi-partner pilots, for example those spanning primary, secondary and social care services, also took longer than anticipated to implement change. Indeed, in some cases, change was only reported towards the end of the two-year pilot period. Sites reported challenges in securing support from stakeholders, each of whom often had their own internal processes and sign-offs needed for key decisions. It is important to note that as part of the national programme, the selection process favoured applications which appeared to have strong project management, and all sixteen Integrated Care Pilots received specific project management support from a firm of management consultants and took part in regular evaluation learning events. So we anticipate that other initiatives might face greater barriers in establishing effective management processes.

#### Information technology

Different IT systems in partner organisations caused difficulties in data-sharing and communicating, especially across health and social care teams. On occasions, these difficulties were not caused by the IT itself but by how their introduction was managed. For example, instead of reducing duplication of effort, poor implementation might increase it:

*So that’s a big issue with [nurses] at the moment, because we are having to triplicate our work really. We’re having to put it on [name of IT system], we’re having to write it in the patients’ notes and then we’re having to go to the GP.* Healthcare professional 1, site 04, Interview

Additionally, and again not a problem inherent to the IT, some partner organisations (most often GP practices) had privacy concerns and were reluctant to share patient data

*We have developed a data sharing agreement which we’re about to test, but it’s just really cumbersome because they want to have it in place for everything. There’s no blanket approval that they can give, so every time you want to share data you’ve got to fill the data sharing agreement in and get it approved, and there’s 16 practices to do that with every time.* Manager 1, Site 04, interview

For one pilot, barriers to data-sharing proved critical. Their original ambition had been to create a shared record which would include data from multiple providers. Despite apparent engagement and high level commitment from the two government departments, the necessary agreement to share data between health and social care was never forthcoming. This site also attempted to develop a SharePoint intranet accessible to all partners, but then discovered that Microsoft’s licensing policy would not allow access to the intranet site, unless an ‘inter-connector’ licence was purchased for which no resources had been allocated. At the end of the pilot period, the site was still attempting to work around these unanticipated problems.

### Relationships and communication

Staff often attributed success to good existing relationships between individuals and/or organisations. Where this was absent, we found that pilots reported substantial challenges in engaging individuals from large numbers of professional groups within a relatively short timeframe. Overcoming these challenges involved clear communication about the contributions required from different participants and the rules governing how the partnership should work. Building relationships was more difficult to obtain in sites where there was disagreement over the benefits of the proposed intervention. By contrast, widespread agreement and shared values among participating staff promoted engagement and motivation. Finally, success was more often reported in pilots where individuals were confident that senior management or team leaders were strongly committed to implementing lasting change.

However, even where there were good pre-existing relationships, almost all sites stressed the importance of ongoing, planned communication between senior executives in the partner organisations.

Co-location was also important in developing relationships. Sites aiming to create a new integrated team found that working together face-to-face in the same building improved the quality and frequency of communication, and expedited problem-solving by allowing quicker access to colleagues’ professional knowledge.

### Professional engagement and leadership, credibility, and shared values

Several sites underestimated the difficulty of securing professional engagement across their whole pilot area, and this presented a barrier to implementation. Sometimes a particular professional group felt sidelined, or uninvolved with planning from the beginning.

*So what they did do across the county? They started a model of integrated teams, which only means …… OTs, physios, nurses, operating in a more integrated way. Where is social care in that? So what is integration?* Social care manager, site 01, interview

*And in terms of that, if I was being overly critical, not about [the Pilot] but the model, I don’t think it’s embracing social care particularly because it’s about a ‘ward’, and people initially think of clinicians.* Social care professional, site 04, interview

In other cases, staff felt de-motivated when there was an absence of clear and consistent communication from leaders within organisations about what work was required and contribution needed from participants. Some staff described feeling thrown into the pilots without having enough preparation on what it would entail and who in management was involved. Staff were also reluctant to engage when there was uncertainty about what individuals were allowed to do:

*Some core team members have been hesitant .... probably the biggest factor we have experienced is the ‘Do we really have permission to do this?’ factor. With this being a multi-organisational project where the work that people do is so visible not only to professionals from their own organisation but to others also, some team members appear to be rather cautious …. so service leads and senior management have been asked to spread the message that the teams have full permission to implement changing practices and are encouraged and supported in doing so.* Living Document, site 01

Where GPs as a group were reluctant to engage, this was described as a major, if not the strongest barrier, to progress. The importance of GP engagement may be particularly evident in the sixteen sites we studied as many were specifically designed from the outset to be GP-led. Commitment from individual GPs was necessary for the credibility of many pilots and where this was lacking, this could be an insurmountable barrier to progress. One site described, ‘ongoing inertia and cynicism’ which resulted in much time devoted to convincing clinicians of the potential benefits of a new integrated service.

Where staff (including GPs) felt the change was being forced upon them, they were less likely to support the new activity. Creating shared beliefs about the benefits of change was described by staff as critical to progress. In successful sites, staff expressed strong support for, and belief in, the pilot’s work, although we cannot be sure whether this was cause or effect. Where a sense of vision was not widespread, progress was noticeably slower, and the barriers cited by staff were greater. One site explained in a Living Document update:

*The former Chief Executive of the Trust engaged his workforce with a very easy to understand vision. He then enabled the people on the frontline to feel involved in changing the services to ensure that they were most effective. This autonomy and motivation really helped translate a vision into relevant changes and service redesign.* Living Document, site 06

Both senior and team leadership were viewed as critical success factors by staff: ‘good’ leadership was commonly identified as facilitating success and ‘poor’ leadership blamed for lack of progress. A few staff members identified a ‘champion’ within teams; someone who reminded colleagues of the project’s benefits and provided sustained motivation. Sometimes this role was held by the project manager or a senior clinical manager, but clinical leadership appeared critical to success in many sites. This was mainly because of the ability of clinicians to engage with and motivate their professional peer group.

Some interventions required clinicians to adopt new responsibilities that were outside their existing roles (e.g., transferring responsibility for initial assessment away from social workers and towards a generic community care worker) or to abandon old ones. The creation of new generic roles sometimes led to an erosion of professional identity. Some staff found that, in creating a team that carried out new types of assessment, their previously ‘owned’ roles and even favourite tasks were lost.

*It is a bit mixed, some people really don’t want to take on generic skills and say they’ve gone into this position because they were doing ‘x, y and z’ and weren’t expected to do the other things that have now come on board.* Healthcare professional 2, site 01, interview

*Perhaps it’s the old values of certain members of staff who don’t like… they see it as… they’ve got their own little role, and they don’t like to see it spreading out to other people, if that makes sense. You know, that’s their job and that’s it, type thing, and they’re a little bit precious about it, rather than divulging it out to other people.* Healthcare professional 1, site 04, interview

The provision of training specific to the service change was important, particularly when the work involved required new or changed roles of participants. Correspondingly, a lack of training sometimes led to staff being unclear whether they were permitted to take on particular tasks or feeling unprepared to take on new roles:

*I think it could have been better planned. There could have been a bit more training in place… Our manager and social workers have supported us, you know, but I think higher up the scale there perhaps could have been a bit more thoughts on training*. Social care professional, site 01, interview

Professionals therefore need to be engaged, provide leadership and develop new skills. In this professionally-led set of processes there is a further tension to manage: the difference between professional and patient perspectives. For example, one Pilot aimed to increase the number of people dying in their place of choice, which was presumed to be at home. However, what had not been anticipated was that the ‘place of choice’ for many patients was hospital rather than home. In an accompanying paper [17], we report quantitative data which shows a mixture of positive and unexpected negative findings that suggest that professional and patient views were not always fully aligned in the new models of care being introduced in the pilots.

### Contextual factors

Staff discussed a number of practical barriers to integrating care which related to the organisational and policy contexts within which the interventions were implemented. These included elements of NHS bureaucracy, regulations governing budgets and employment in different care sectors, external reforms, concurrent internal reorganisation, staff turn-over, and organisational culture.

#### Public service bureaucracy

A common frustration expressed by sites was the perception of unnecessary delays when getting new activities started. Some of the practicalities of health service bureaucracy—chains of managerial approval among multiple organisations and slow decisions about resource distribution—were perceived as cumbersome and a barrier to innovation.

*I do think there are some occasions where things take an inordinate amount of effort to get done, much more than I expected, to be honest.* Manager 1, Site 04, Interview

In addition, there were difficulties when planned changes in the delivery of care encountered human resources or legal issues within partner organisations. Several sites faced problems in pooling budgets that historically had been used for either health or social care, and found money was often tied up within that organisation’s spending plans or that use of resources was constrained by the organisation’s own regulations. Some sites commented that NHS financial regulations prevented partners from establishing a ‘whole system of care’ (e.g., by merging budgets). Substantial delays also occurred where changes to staff employment were involved. In one case, the national Co-operation and Competition Panel had to rule on the legality of changes proposed in the pilot, and this prevented progress of the originally intended activity.

#### Resources allocated to the pilot

Issues surrounding financial resources allocated to Pilots were a substantial issue. Funding was important and was in theory provided in order to backfill staff seconded to work in the pilot. However, staff often found that they had to complete activities relating to the pilot on top of their existing workload. The personal cost to staff of having to take on more work for potential patient benefit at some time in the future created a difficulty in developing motivation.

*Yes, it’s the investment of time as well, not necessarily the money, but just having an open mind to try something different and being prepared to perhaps have to put in a bit of effort at the front end to reap the benefits at the end. And I think that’s difficult for staff at the moment when they’re struggling to keep on top of their existing workload… a leap of faith.* Manager 1, Site 04, interview

Another site commented through a Living Document:

*Things only progress when the key people involved in the project push things forward and these key people are doing other jobs as well. The need to use internal resources who are already committed to full-time jobs is a key inhibiting factor for delivering any change in the NHS.* Living Document, site 11

Staff in some of the sites spoke of working overtime in order to keep pilot activity going and raised questions about the sustainability of such activity in the longer term.

In addition to financial resources, pilot sites were provided with the services of a firm of management consultants as part of the national programme to support Pilot implementation. This included a structured approach to the identification of risks which might compromise the Pilots progress. Despite this risk assessment exercise, sites found it difficult to respond to factors which compromised progress of the pilot when they occurred.

Delivery of planned changes to care was also seriously affected by financial pressures on the NHS which developed during the course of the Integrated Care Pilot evaluation. For example, one site lost four key staff posts (six community matrons reduced to three and three community service advisers reduced to one) which they believed were critical to running community wards. In other cases, local authority budgets were cut and fewer social workers than anticipated were available for the ICP. The development of additional financial constraints during the course of the pilots led to a change in attitude, with staff increasingly viewing pilots as successful if they reduced costs, rather than meeting the original objectives which were more often focused on clinical outcomes. However, wider financial constraints were also sometimes seen as supporting the proposed changes by raising the importance of integration and the need to change working practices. This was sometimes referred to by the Pilots as the ‘burning platform’ providing a spur to action.

*It is the only way forward in managing and providing services if we wish to reduce waste and patient frustration.* Living Document, Site 09

#### External policy reform

Other national-level changes had a mixed effect on progress of the pilots. For example, a national strategy designed to integrate health and community services, known as Transforming Community Services [18] was introduced during the evaluation period. This was perceived as actively helpful in implementing some pilot activity by clarifying relationships with community services. However, for other pilots, the same national policy inhibited change, as the policy required a clear separation between commissioning and providing functions—in at least one Pilot the aim was to achieve closer integration between these two functions.

#### Organisational culture

Delivery of the pilots’ objectives relied on their ability to modify existing systems and practices and to create new ones. This was especially dependent on organisational culture which included local perceptions of professional boundaries. New management structures of integrated teams felt foreign to some staff members who were accustomed to more ‘silo-type working’, e.g., where physiotherapists always manage the physiotherapists; district nurses are led by a nurse, etc.

In addition to needing to renegotiate professional boundaries, staff often found activities were hampered by a lack of openness which was part of a wider NHS ‘blame culture’:

*The time required to build relationships and trust, to enable frank open and constructive discussions to take place without feelings of blame and attribution…. as a newcomer to the NHS the blame culture seems to be strong, particularly across organisational boundaries.* Living Document, site 11

In contrast, two sites specifically commented that their pilot had enabled people to move away from such a ‘blame culture’ and thus make a new culture possible:

*I personally would say that the partnership has achieved considerable success in its short existence especially around increased knowledge of whole-system challenges and opportunities, promoting a no-blame culture and developing a ‘we are all in it together’ mantra.* Living Document, site 03

*We have created a group called Transforming Integrated Care which holds monthly meetings and has representatives of managers from all four organisations. This is again unique and allows mature conversations to be held in a constructive manner, moving away from the silo/blame culture that sometimes impedes such discussions.* Living Document, site 06

One site reported that an external facilitation had been very helpful in getting two organisations to work together. It is worth reiterating that these approaches to improving joint working and organisational culture are well rehearsed in the wider literature on managing change.

## Discussion

The nature of the planned interventions varied across the sixteen sites, including some large-scale infrastructure changes along with more modest adaptations to ways of working. Broadly, the larger and more complex the intervention, the more difficult it was to implement the desired changes. This is unsurprising but was not always taken sufficiently into account in establishing the capacity for delivery and management. Values and professional attitudes were important facilitators, with shared values, collectively shared and well communicated vision, and clinician-led efforts to achieve widespread staff engagement cited as particularly important. Where the participation of key staff groups was assumed rather than actively pursued (e.g., GPs), it was difficult to make progress. It was much easier to make progress where staff could see clear benefits that would result from the changes proposed and where they felt actively involved. Changing roles presented particular challenges for staff, especially where individual staff roles or professional identities were threatened. This was often seen to be a barrier to change. It was also apparent that education and training specific to the new role increased the chance of successful engagement by staff, though we do not know if this was because of the technical skills acquired through training or as a result of more normative adaptations to the new role. Policies and practices relating to IT use were often cited as barriers to communication and data exchange. Other barriers identified included changes to national policies and current financial and employment structures of health and social care in England. Having arrived at these conclusions, we then examined how these results compared with other studies of change in health care and whether our findings supported or undercut previous analyses. This was not intended to be a reinterpretation of our data but to provide a way of interpreting our data in the light of a wider evidence base.

First, we found that our findings are consistent with previous studies which stress the importance of clear goals, effective leadership, integrated data systems, common assessment procedures and joint training when planning integrated care [19, 20] and studies which identify the importance of shared values, shared understanding of roles, shared education and good communication for effective multi-disciplinary team functioning [21, 22]. Furthermore, the results are also consistent with studies looking at barriers and facilitators of improvement in healthcare more generally, for example a recent systematic review of quality improvement by Kaplan et al. [14], which categorises elements essential to success as listed in Table 1. We compared our findings with those of Kaplan et al. The results are summarised in Table 1 which maps each category identified by Kaplan and colleagues against the barriers and facilitators reported by staff in our evaluation. As can be seen in Table 1, our findings add depth to the generic conclusions of Kaplan et al. but broadly reinforce their findings.

Kaplan et al. [14] conclude that mixed success among quality improvement initiatives is most likely due to differences in contextual factors surrounding these initiatives, a conclusion which is also compatible with our own findings.

Our findings therefore contribute to analyses of the organisational facilitators and barriers to change. In addition, because delivering more integrated care may also require significant change in how staff and service users work with each other, we also interpreted our findings in the context of research on how new working practices might become normalised—specifically, why and how people change what they actually do in response to an innovation or project, such as integrated care. In Normalisation Process Theory, May and colleagues [15, 16] seek to explain how complex interventions become routinised in health care practice. They also recognise that normalisation is not an inevitable outcome of collective action; intended values and behaviours may not always become normalised. The authors present four major activity themes necessary for a planned change to become embedded in every day activity, and these are shown in Table 2. As in Table 1, we present the evidence from the Pilots alongside barriers and facilitators suggested by Normalisation Process Theory.

Neither Kaplan nor May claim to offer a complete and overarching theory of delivering change in health care systems, but each offers a set of mid-range conceptualisations and observations, and each can be assessed against its ability to explain known evidence. We have also drawn on May et al. to consider how new forms of working become normalised with attention to the micro-level, although this would be shaped by many of the meso-level organisational factors considered by Kaplan and colleagues. For example, both would consider the relevance of leadership and communication but for NPT this is viewed more from the perspective of employees engaging in and understanding the project and changing their behaviour (or not). The interventions developed across these sixteen pilots almost exclusively operated at the meso-level (i.e., organisational level), but many of the barriers and facilitators we found related to the micro (i.e., individual) level.

Kaplan and May’s approaches are oriented towards different dimensions of change. Kaplan’s framework allows us to consider impacts of contextual factors, such as leadership, infrastructure and prior relationships, while May and colleagues point to the factors associated with individual motivation and change. Clearly, issues such as effective communication overlap, but when taken together, these two frameworks reveal a developing set of factors that can be used both to explain relative success and failure of change efforts in health care, and also provide a ‘checklist’ for issues to be attended to by those seeking to deliver such changes. Together, the elements of quality improvement presented by Kaplan et al. [14] and the Normalisation Process Theory developed by May et al. [15, 16] provide a helpful way to think about and communicate our findings. They also provide some issues to be further explored in future work. In summary, these findings highlight the importance of senior leadership; team leadership; a facilitating organisational culture; data infrastructure and information; previous staff involvement in quality improvement initiatives; physician involvement; adequate resources; and the local circumstances shaping context.

Although facilitators and barriers to success of the pilots studied varied across the different sites, the recurrent themes discussed here—many similar to those of wider quality improvement initiatives—may be useful to policymakers and organisations looking to integrate health and/or social care services. Delivering integrated care requires multiple preparatory activities, perhaps most importantly, clear, effective communication across different organisations, service users, staff groups and professions. Participating staff not only want to understand their role in the new arrangement but how this role will fit with their current professional identities and their own personal development. This understanding underpins the need for routine embedding of an intervention in everyday practice, i.e., ‘normalisation’ [15]. The findings of this study reinforce those of Ham et al. [23] but don’t necessarily support their conclusion that ‘dogged attention to project and change management’ is required. Because of a policy imperative to produce results, these sites were provided with extensive project management support from an external management consultant but this did not seem to prevent the barriers that others have encountered. One possibility is that too much was expected of the sites in the two-year pilot period. A recent report on an experiment to integrate care found that “two years of initial development followed by one year of live working” was required to show significant change [24].

**Figure 1.**
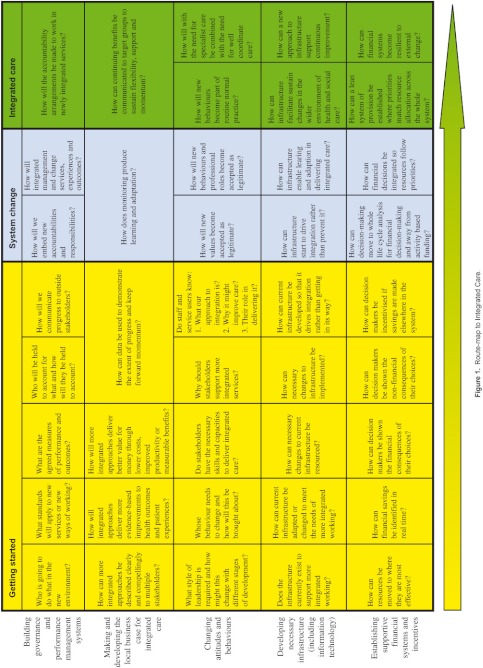
Route-map to Integrated Care.

Taken together we can see that delivering integrated care requires a balance of activities which attend to motivational and cultural as well as organisational and infrastructural factors. We suspect, but did not test here, that sustained progress towards more integrated forms of delivery requires a judicious balance across all of these dimensions over time, although it also seems likely that in the short run progress can be made by driving forward on just one front. If correct, this is important for decision makers because this suggests that what is required to make early ‘quick wins’ may need to be modified to produce more sustained change. Conversely, approaches which appear to make limited progress in terms of measurable outcomes in the short term might be putting in place a balanced platform for future delivery. Drawing on the detailed lessons from the pilots, as reinforced by more generic literature, we identified a set of activities which need to be considered by those planning to provide better integrated care. These are presented in the form of a routemap (Figure 1). This is not a guide to providing integrated care, rather a guide to the key questions which need to be considered as part of the change process.

## Conclusion

In conclusion, we have described the facilitators and barriers associated with delivering the Integrated Care Pilots, based on the perceptions and experiences of those most closely involved. We have seen that these conclusions are compatible with previous findings. Many barriers and facilitators to integrating care are those of any large-scale organisational change including issues relating to leadership, organisational culture, information technology, physician involvement, and availability of resources. However, activities which appear particularly important for delivering integrated care include personal relationships between leaders in different organisations, the scale of planned activities, governance and finance arrangements, support for staff in new roles, and organisational and staff stability. We illustrate our analyses with a ‘routemap’ which identifies questions that providers may wish to consider when planning interventions to improve the integration of care.

## Figures and Tables

**Table 1:**
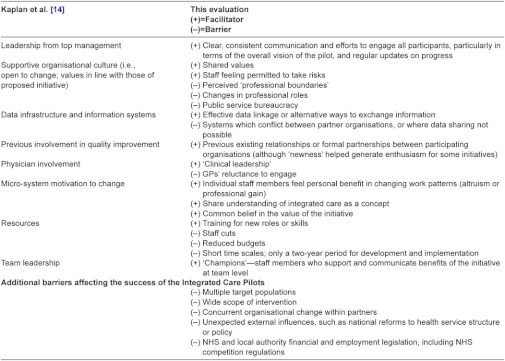
Factors found to be associated with success in quality improvement and integrated care initiatives.

**Table 2:**
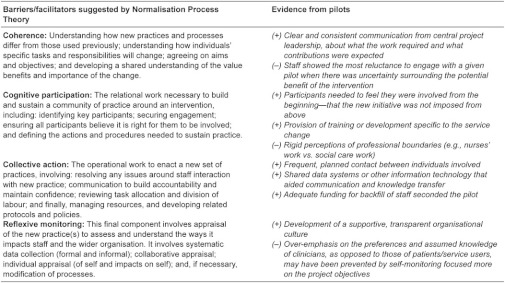
Four categories described in Normalisation Process Theory followed by examples of facilitators and barriers from the Integrated Care Pilot evaluation.

## References

[1] Yach D, Hawkes C, Gould CL, Hofman KJ (2004). The global burden of chronic diseases. The Journal of the American Medical Association.

[2] Glasby J, Dickinson H, Peck E (2006). Partnership working in health and social care. Health and Social Care in the Community.

[3] Bodenheimer T, Wagner EH, Grumbach K (2002). Improving primary care for patients with chronic illness. The Journal of the American Medical Association.

[4] Bodenheimer T, Wagner EH, Grumbach K (2002). Improving primary care for patients with chronic illness: the chronic care model, Part 2. The Journal of the American Medical Association.

[5] Nolte E, McKee M (2008). Caring for people with chronic conditions: a health system perspective.

[6] Department of Health (2008). Next Stage Review. Our vision for primary care.

[7] Department of Health (2012). Integrated Care Pilots: an introductory guide.

[8] Romøren TI, Torjesen DO, Landmark B (2011 Oct 7). Promoting coordination in Norwegian health care. International Journal of Integrated Care [serial online].

[9] RAND Europe, Ernst, Young LLP (2012). National evaluation of the DH integrated care pilots. Appendix G. Overview of Integrated Care Pilot sites.

[10] RAND Europe, Ernst and Young LLP (2012). National evaluation of the DH integrated care pilots. http://www.rand.org/randeurope/research/projects/integrated-care-pilots.html.

[11] Powell Davies G, Williams AM, Larsen K, Perkins D, Roland M, Harris M (2008). Coordinating primary health care: an analysis of the outcomes of a systematic review. Medical Journal of Australia.

[12] Ling T, Bardsley M, Adams J, Lewis R, Roland M (2010). Evaluation of UK Integrated Care Pilots: research protocol. International Journal of Integrated Care [serial online].

[13] Dixon-Woods M, Agarwal S, Jones D, Young B, Sutton A (2005). Synthesising qualitative and quantitative evidence. Journal of Health Service Research Policy.

[14] Kaplan HC, Brady PW, Dritz MC, Hooper DK, Linam WM, Froehle CM (2010). The influence of context on quality improvement success in health care: A systematic review of the literature. Milbank Quarterly.

[15] May C, Finch T, Mair F, Ballini L, Dowrick C, Eccles M (2007). Understanding the implementation of complex interventions in health care: the normalization process model. BMC Health Services Research.

[16] Murray E, Treweek S, Pope C, MacFarlane A, Ballini L, Dowrick C (2010). Normalisation process theory: a framework for developing, evaluating and implementing complex interventions. BMC Medicine.

[17] Roland M, Lewis R, Steventon A, Abel G, Adams J, Bardsley M (2012). T Case management with predictive risk modelling in the English integrated care observational study of staff and patient experience, secondary care utilisation and costs. International Journal of Integrated Care [serial online].

[18] Department of Health (2009). Transforming community services: enabling new patterns of provision.

[19] MacAdam M (2008). Frameworks of integrated care for the elderly: a systematic review. CPRN Research Report 2008.

[20] McAdam M (2011). Progress toward integrating care for seniors in Canada. International Journal of Integrated Care [serial online].

[21] Hjelmar U, Hendriksen C, Hansen K (2011). Motivation to take part in integrated care—an assessment of follow-up home visits to elderly persons. International Journal of Integrated Care [serial online].

[22] Cramm JM, Nieboer AP (2011). Professionals’ views on inter-professional stroke team functioning. International Journal of Integrated Care [serial online].

[23] Ham C, Parker H, Singh D, Wade E (2008). Making the shift from hospital to the community: lessons from an evaluation of a pilot programme. Primary Health Care Research and Development.

[24] Shaw S, Levenson R (2011). Towards integrated care in Trafford.

